# Selective potentiation of 2-APB-induced activation of TRPV1–3 channels by acid

**DOI:** 10.1038/srep20791

**Published:** 2016-02-15

**Authors:** Luna Gao, Pu Yang, Peizhong Qin, Yungang Lu, Xinxin Li, Quan Tian, Yang Li, Chang Xie, Jin-bin Tian, Chengwei Zhang, Changlin Tian, Michael X. Zhu, Jing Yao

**Affiliations:** 1 Key Laboratory of Molecular Biophysics of the Ministry of Education, College of Life Science and Technology, Huazhong University of Science and Technology, Wuhan, Hubei 430074, China; 2 College of Life Sciences, Wuhan University, Wuhan, Hubei 430072, China; 3 Department of Integrative Biology and Pharmacology, The University of Texas Health Science Center at Houston, Houston, TX 77030; 4 Hefei National Laboratory of Microscale Physical Sciences, School of Life Sciences, University of Science and Technology of China, Hefei, Anhui 230026, China

## Abstract

Temperature-sensitive TRP channels are important for responses to pain and inflammation, to both of which tissue acidosis is a major contributing factor. However, except for TRPV1, acid-sensing by other ThermoTRP channels remains mysterious. We show here that unique among TRPV1–3 channels, TRPV3 is directly activated by protons from cytoplasmic side. This effect is very weak and involves key cytoplasmic residues L508, D512, S518, or A520. However, mutations of these residues did not affect a strong proton induced potentiation of TRPV3 currents elicited by the TRPV1–3 common agonist, 2-aminoethoxydiphenyl borate (2-APB), no matter if the ligand was applied from extracellular or cytoplasmic side. The acid potentiation was common among TRPV1–3 and only seen with 2-APB-related ligands. Using ^1^H-nuclear magnetic resonance to examine the solution structures of 2-APB and its analogs, we observed striking structural differences of the boron-containing compounds at neutral/basic as compared to acidic pH, suggesting that a pH-dependent configuration switch of 2-APB-based drugs may underlie their functionality. Supporting this notion, protons also enhanced the inhibitory action of 2-APB on TRPM8. Collectively, our findings reveal novel insights into 2-APB action on TRP channels, which should facilitate the design of new drugs for these channels.

Four members of the transient receptor potential vanilloid (TRPV) family, TRPV1–4, have been implicated to play important roles in mammalian chemo-somatosensing, such as changes in temperature, pressure, and osmolarity, as well as a large number of endogenous and exogenous chemical activators, including inflammatory mediators^1^,^2^. Indeed, a pivotal role for TRPV1 in sensing noxious heat and mediating thermal and inflammatory pain has been well established^3^. However, whether or not and to what extent other closely related TRPV channels are involved in similar physiological functions like TRPV1 remain debated^4^,^5^. All four TRPV channels respond to temperature increases with TRPV3 and TRPV4 being activated at warm and innocuous temperatures while TRPV1 and TRPV2 by noxious heat. The different temperature thresholds for channel opening and the temperature ranges these channels act at are particularly interesting, giving rise to the idea that TRPV1–4, together with the cold sensing TRPA1 and TRPM8 channels, serve as molecular thermometers (“thermoTRPs”) in mammals^1^,^5^,^6^. However, temperature is only one of the multiple modalities that modulate TRP channel activities; chemical mediators can also strongly affect TRPV channels either through direct ligand binding or signaling pathways that result in posttranslational modifications, such as phosphorylation^7^,^8^.

Tissue acidosis represents a noxious condition associated with infection, inflammation, ischemia, cancer and defective acid containment^9^. The drop in extracellular pH (acidification) alters the activity of many ion channels and receptors. It has been well recognized that acidic solutions activate TRPV1 channels and lower its temperature threshold for heat activation^10^. Critical amino acid residues involved in proton sensing are localized at extracellular loops of the TRPV1 protein^11^,^12^. However, extracellular protons also exert an inhibitory effect on single channel conductance of TRPV1 via two negatively charged glutamate residues at the pore loop^13^,^14^. For other thermoTRPVs, extracellular acid activated TRPV4 expressed in Chinese hamster ovary (CHO) cells^15^ but inhibited a TRPV4-like response in mouse esophageal epithelial cells^16^. TRPV2 was reportedly insensitive to acid^12^,^15^. TRPV3 was initially found not to be activated by extracellular acid^17^ but more recently shown to be potently activated by intracellular acidification induced by glycolic acid^18^. Site-directed mutagenesis revealed that an N-terminal histidine residue, H426, known to be critical for TRPV3 activation by its synthetic ligand, 2-aminoethoxydiphenyl borate (2-APB)^19^, might be involved in sensing the intracellular pH drop. However, the H426N mutation only showed a partial loss of proton activation^18^, indicating that additional amino acids could also participate in acid activation of TRPV3. Similar to TRPV1, acid also appeared to inhibit TRPV3, but the mechanism remains unknown^18^.

TRPV3 has temperature thresholds ranging from 31 to 39 °C^17^,^20^,^21^. However, to what extent TRPV3 contributes to body’s temperature sensing is uncertain. TRPV3 knockout (KO) mice exhibited impaired temperature sensing and thermal preference^22^, but these phenotypes might depend on the genetic backgrounds^5^. On the other hand, the channel appears to have prominent functions in the skin. TRPV3 is predominantly expressed in epidermal keratinocytes, especially to the outer root sheath of epithelial compartments of hair follicles, and epithelial layer of the nose and mouth^22^,^23^,^24^. TRPV3 KO mice display wavy hair coat and curly whiskers and have defects in skin barrier formation because of the impaired keratinocyte cornification^25^. Also interesting is that up-regulation of TRPV3 function impairs hair growth and increases the incidence of dermatitis and pruritus in both humans and rodents, suggesting that either too much or too little TRPV3 activity is detrimental to skin health^26^,^27^,^28^. Although several exogenous compounds, such as 2-APB, some flavor additives, skin sensitizers and allergens, activate TRPV3^24^,^29^,^30^, the endogenous TRPV3 activators are yet to be determined.

Given the importance of maintaining proper TRPV3 function to skin health and the inevitable exposure of TRPV3-expressing keratinocytes to acidic pH under physiological conditions of skin, which has a mean pH around 5.5 (ranging from 4 to 7)^31^, it is critical to understand how TRPV3 channels respond to pH changes. Here, we show that TRPV3 is weakly activated by protons only from the intracellular side, but low pH strongly potentiates the response to 2-APB of not only TRPV3 but also TRPV2 and TRPV1 no matter if the ligand was applied from extracellular or cytoplasmic side. The proton activation and potentiation employed different mechanisms, with residues in the S2–S3 loop required in the activation and acid-induced structural changes of the boron-containing compound likely involved in the ligand-dependent potentiation.

## Results

### Acid potentiates the responses of TRPV3 and TRPV2 channels to 2-APB

**Figure 1:**
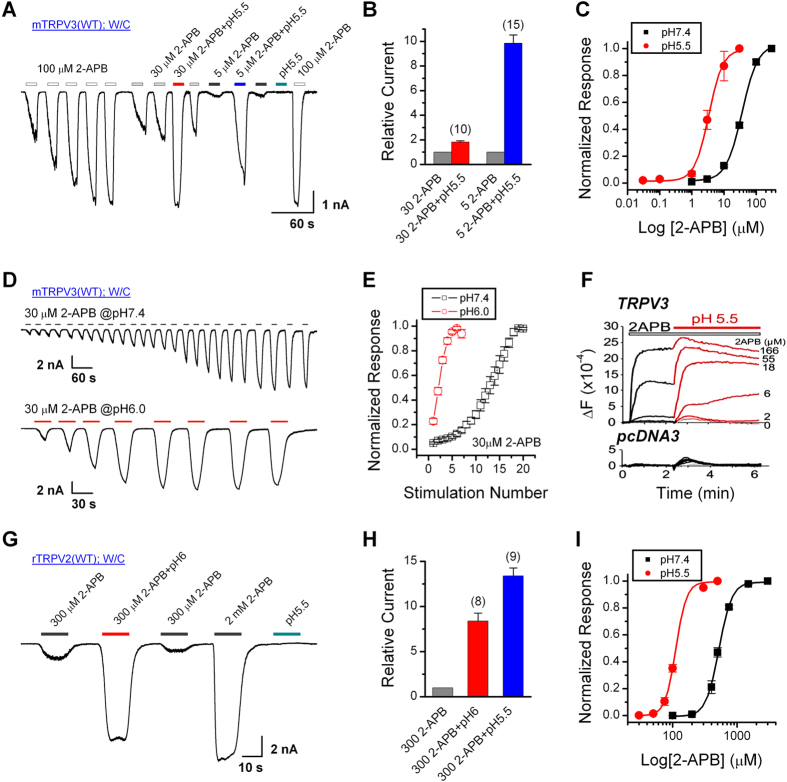
Acid enhances the activity of TRPV3 and TRPV2 channels expressed in HEK293 cells. (**A**) Acid potentiates 2-APB response in a representative TRPV3-expressing HEK293 cell. After sensitization by repeated application of 100 μM 2-APB, the cell was exposed to 30 or 5 μM 2-APB without or with the pH lowered to 5.5. (**B**) Summary of relative currents elicited by 30 and 5 μM 2-APB at pH 7.4 and pH 5.5. Currents were normalized to that evoked by the same concentration of 2-APB at pH 7.4. Numbers of cells are indicated in parentheses. (**C**) Concentration-response curves of 2-APB at pH 7.4 and pH 5.5 after full sensitization of TRPV3. The solid lines are fits to the Hill equation with EC_50_ = 36.2 ± 2.1 μM and n_H_ = 1.76 ± 0.17 for pH 7.4 (*n* = 8) and EC_50_ = 3.34 ± 0.29 μM and n_H_ = 1.98 ± 0.34 for pH 5.5 (*n* = 9). (**D**) Representative whole-cell recordings for sensitization of TRPV3 currents evoked by repeated applications of 30 μM 2-APB at pH 7.4 and pH 6.0. (**E**) Time courses of peak currents elicited by repeated applications of 30 μM 2-APB at pH 7.4 (n = 6) and pH 6.0 (n = 8). Currents were normalized to the maximum value after sensitization. (**F**) [Ca^2+^]_i_ increases elicited by different concentrations of 2-APB measured by Fluo-4 in TRPV3 (*upper*) and pcDNA3-transfected cells (*lower*). The extracellular pH was 7.4 initially and subsequently lowered to 5.6 without a change in 2-APB concentration. Measurements were performed at 32 °C with 1.8 mM extracellular Ca^2+^. (**G**) Representative whole-cell currents at −60 mV in a TRPV2-expressing HEK293 cell treated with 300 μM and 2 mM 2-APB at pH 7.4, 300 μM 2-APB at pH 6.0, and pH 5.5 only. (**H**) Summary of relative currents elicited by 300 μM 2-APB at pH 7.4, 6.0 and 5.5. (**I**) Concentration-response curves of 2-APB at pH 7.4 and pH 5.5. EC_50_ = 522.2 ± 10.2 μM and n_H_ = 4.34 ± 0.35 at pH 7.4 (*n* = 11); EC_50_ = 112.2 ± 1.2 μM and n_H_ = 5.26 ± 0.35 at pH 5.5 (*n* = 7). Error bars represent SEM.

We first examined the effect of extracellular acid on 2-APB-evoked TRPV3 currents. Figure 1A illustrates whole-cell currents recorded in HEK293 cells that transiently expressed mouse TRPV3 (Holding potential, V_h_ = −60 mV). Because TRPV3 channels undergo sensitization upon repeated stimulation^21^,^32^, we examined the effect of extracellular acidification after the response had stabilized following repeated applications of 100 μM 2-APB at the neutral pH (7.4). Then, the sensitized cell was exposed to 30 or 5 μM 2-APB at pH 7.4 or pH 5.5. The responses at the lower pH were remarkably larger than at the neutral pH. The peak current amplitudes evoked by 30 and 5 μM 2-APB at pH 5.5 were ~2- and 10-fold, respectively, of that at pH 7.4 (Fig. 1B). Notably, acid alone (pH 5.5) did not elicit any detectable current, suggesting that extracellular acid mainly affected 2-APB-induced TRPV3 activities. Figure 1C shows that the low pH caused a left-shift of the concentration-response curve to 2-APB from the whole-cell experiments, with the EC_50_ reduced by about 12-fold (at pH 7.4: EC_50_ = 36.2 ± 2.1 μM, n_H_ = 1.8 ± 0.2; at pH 5.5: EC_50_ = 3.3 ± 0.3 μM, n_H_ = 2.0 ± 0.3). The above results suggest that extracellular acid reversibly enhances the response of TRPV3 to 2-APB.

We reasoned that by enhancing the response to 2-APB, acid might also accelerate the process of TRPV3 sensitization by repeated stimulation. To examine this possibility, the TRPV3-expressing cells were repeatedly exposed to 30 μM 2-APB at pH 7.4 or pH 6.0 for ~15 s each time and followed by a thorough washout (Fig. 1D). As expected, the lower pH not only enhanced the current amplitude elicited by the first application of 2-APB, but also reduced the number of stimulations required to reach full sensitization (Fig. 1D,E).

TRPV3 forms Ca^2+^-permeable channels that respond to 2-APB stimulation with an increase in intracellular Ca^2+^ concentration ([Ca^2+^]_i_), which can be monitored in microtiter plates in a population of cells loaded with the Ca^2+^ indicator dye, Fluo-4^29^. In TRPV3-transfected cells loaded with Fluo-4 and seeded in a 96-well plate, 2-APB evoked robust fluorescence increases at pH 7.4 in a concentration-dependent manner (Fig. 1F, *upper panel, black traces*). A subsequent reduction of the extracellular pH to 5.5 in the continued presence of 2-APB markedly enhanced the fluorescence intensities in cells exposed to low to moderate 2-APB concentrations (6–55 μM) (Fig. 1F, *upper panel, red traces*). With the high 2-APB concentration (166 μM), the Ca^2+^ response approached maximum and therefore was not further increased by pH 5.5. The small bump observed at 2 and 166 μM 2-APB mostly reflected an endogenous response of HEK293 cells to acid, as it was also observed in vector-transfected control cells whether or not 2-APB was present (Fig. 1F, *lower panel*).

Similar to TRPV3, although TRPV2 was insensitive to acid alone (Fig. 1G), consistent with the previous report^12^, the whole-cell currents in TRPV2-expressing cells evoked by 300 μM 2-APB at pH 6.0 and pH 5.5 were 8-fold and 13-fold, respectively, of that at pH 7.4 (Fig. 1G,H). The concentration-response curve to 2-APB was left shifted by pH 5.5, with the EC_50_ reduced from 522.2 ± 10.2 μM (n_H_ = 4.3 ± 0.3) at pH 7.4 to 112.2 ± 1.2 μM (n_H_ = 5.2 ± 0.3) at pH 5.5 (Fig. 1I). Therefore, extracellular acid also enhances the sensitivity of TRPV2 to 2-APB.

### Extracellular acidic residues in TRPV3 and TRPV2 are not responsible for proton potentiation

Typically, protons regulate protein functions by altering conformations through protonations at glutamate (E), aspartate (D), or histidine (H) residues. In TRPV1, the glutamic residue (E600) on the extracellular side has been identified as crucial for the acid potentiation^11^. To define the mechanism of acid sensing of TRPV3 and TRPV2, we first mutated the equivalent residues, E610 in TRPV3 and E561 in TRPV2, to glutamine (Q) and then examined whether the mutation could alter the acid effect. However, the E610Q mutation of TRPV3 resembled the wild-type (WT) channel in acid potentiation, with the current amplitudes evoked by 30 and 5 μM 2-APB at pH 5.5 being about 2- and 7-fold, respectively, of that at pH 7.4 (Fig. S1A, S1B). We then individually neutralized all extracellular glutamates, aspartates, and histidines in TRPV3 by mutagenesis and tested the low pH-induced changes in currents evoked by 5 μM 2-APB following transient expression of the mutant channel in HEK293 cells. Despite small variations in the fold increase induced by pH 5.5, the acid potentiation remained in all mutant channels (Fig. S1C). Similarly, neutralizing the negative charges in the extracellular side of TRPV2 by mutagenesis also failed to alter the acid potentiation of 2-APB-evoked TRPV2 currents (Fig. S1D-S1F). Overall, the negatively charged residues in the extracellular loops do not appear to be involved in the potentiation by extracellular protons of 2-APB evoked TRPV3 and TRPV2 currents.

### Acid potentiates 2-APB-evoked currents from the cytoplasmic side in TRPV3, TRPV2 and TRPV1

**Figure 2:**
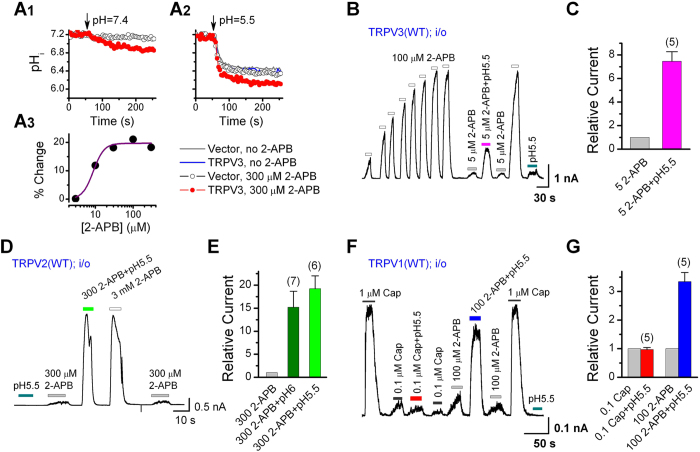
Acid potentiates 2-APB-evoked currents from the cytoplasmic side in HEK293 cells that expressed TRPV3, TRPV2 and TRPV1. (**A**) 2-APB and extracellular acidification lower intracellular pH (pH_i_). Effects of 2-APB (A1) and low extracellular pH (pH 5.5, A2) on pH_i_, measured by BCECF, in control (*open circles*) and TRPV3-expressing cells (*filled circles*). Panel (A3) shows concentration response to 2-APB in TRPV3-expressing cells. The solid line represents a fit to the Hill Equation. (**B**) Representative currents at +60 mV recorded in an inside-out patch excised from a TRPV3-expressing cell. After sensitization by repeated applications of 100 μM 2-APB to the cytoplasmic side, the patch was exposed to 5 μM 2-APB at pH 7.4 and pH 5.5. Note, pH 5.5 alone also elicited a small increase in current when applied from the intracellular side. (**C**) Relative TRPV3 currents elicited by 5 μM 2-APB at pH 7.4 and pH 5.5 from the cytoplasmic side. (**D,E**) Effects of intracellular acid on 2-APB evoked TRPV2 currents in inside-out patches. Representative current trace (**D**) and average responses (**E**) show that lowering pH_i_ to 6.0 and 5.5 strongly enhanced the current elicited by 300 μM 2-APB. Note, pH 5.5 alone did not induce any detectable current. (**F**) Representative TRPV1 currents recorded from an inside-out patch stimulated with varying concentrations of capsaicin (Cap) or 2-APB at either pH 7.4 or 5.5 applied from the cytoplasmic side. Note, when applied intracellularly, pH 5.5 did not induce any measurable TRPV1 current. (**G**) Relative TRPV1 currents at +60 mV elicited by 0.1 μM Cap and 100 μM 2-APB at pH 7.4 and 5.5 from the cytoplasmic side. The acidic pH only potentiated the response to 2-APB but not that to Cap. Membrane patches were excised from TRPV3, TRPV2 or TRPV1-expressing HEK293 cells. Holding potential was +60 mV. Error bars represent SEM.

The above experiments suggest that extracellular acidification indeed enhances channel activities of TRPV3 and TRPV2, but the acidic residues at the extracellular sides of these channels are unlikely to be responsible for the acid potentiation. It was recently demonstrated that acid strongly activated TRPV3 from the cytoplasmic side^18^. We therefore tested the possibility that the extracellularly applied acid potentiated the TRPV channel function through acidification of the intracellular environment. First, we measured intracellular pH changes induced by extracellular acidification using the fluorescence pH indicator, BCECF. In BCECF-loaded HEK293 cells, lowering the extracellular pH from 7.4 to 5.5 quickly decreased the BCECF fluorescence ratio (F_490_/F_440_) (Fig. 2A), indicating that extracellular acidification induced a drop in intracellular pH in HEK293 cells. The degree of the decrease was similar between the control and TRPV3-expressing cells, suggesting that the intracellular pH drop was independent of TRPV3 expression. Interestingly, the BECEF ratio also moderately decreased in response to application of 2-APB (300 μM) without a change of the extracellular pH, indicative of intracellular acidification by 2-APB alone (Fig. 2A1). This effect was very weak in the control HEK293 cells, but stronger in cells that stably expressed TRPV3, with an estimated EC_50_ of 8.8 ± 1.5 μM (Fig. 2A3). Application of 2-APB with the low pH solution induced a further decrease in the BCECF ratio than the low pH alone (Fig. 2A2), but the effect appeared to be additive and still dependent on TRPV3 expression. Therefore, although 2-APB may cause a moderate pH drop in the cytosol via activating TRPV3, this does not synergize with the common, TRPV3-independnt, pathway through which extracellular protons enter HEK293 cells.

To determine whether acid enhances TRPV3 channel function from the cytoplasmic side, we carried out inside-out recordings in TRPV3-expressing HEK293 cells. After sensitization induced by repeated application of 100 μM 2-APB, the patch was exposed to 5 μM 2-APB at pH 7.4 or pH 5.5 (Fig. 2B). We found that the currents at pH 5.5 were about 8-fold of that at pH 7.4 (Fig. 2C). Noticeably, in the absence of 2-APB, pH 5.5 alone also elicited very small, but clearly detectable, currents when applied from the cytoplasmic side (Fig. 2D).

Similarly, inside-out patches excised from cells expressing WT TRPV2 also showed acid potentiation from the intracellular side (Fig. 2D). Currents evoked by 300 μM 2-APB at pH 6.0 and pH 5.5 were approximately 15-fold and 19-fold, respectively, of that at pH 7.4 (Fig. 2E). However, acid (pH 5.5) alone did not elicit any detectable current in these patches. On the other hand, the intracellular acid did not enhance capsaicin-evoked currents in inside-out patches excised from TRPV1-expressing cells (Fig. 2F,G). Neither did the pH 5.5 solution elicit any measurable current on its own (Fig. 2F). However, we surprisingly found that the acidic pH (pH 5.5) still augmented the peak current evoked by 2-APB (100 μM) in patches excised from TRPV1-expressing cells, causing more than 2-fold increase over the 2-APB-induced currents at pH 7.4 (Fig. 2G). These data suggest that intracellular protons specifically potentiate TRPV1 currents evoked by 2-APB, but not that by the selective TRPV1 agonist, capsaicin.

### S2–S3 linker is critical for proton activation of TRPV3

The above results suggest at least two forms of regulation of TRPV1–3 channels by intracellular protons: 1) direct activation, albeit very weak, specific to TRPV3 and 2) potentiation of 2-APB-induced currents, common to all three TRPV channels. It was unclear whether the two reflected separate acid-induced effects on the channels or just different aspects of the same acid-sensing mechanism. Previously, a mutation at an intracellular residue of TRPV3 (H426N) was shown to partially abrogate the proton-evoked channel activation^18^. Because, the same mutation had also been shown to impair TRPV3 activation by 2-APB^19^, acid and 2-APB sensing of TRPV3 was thought to share a common mechanism.

**Figure 3:**
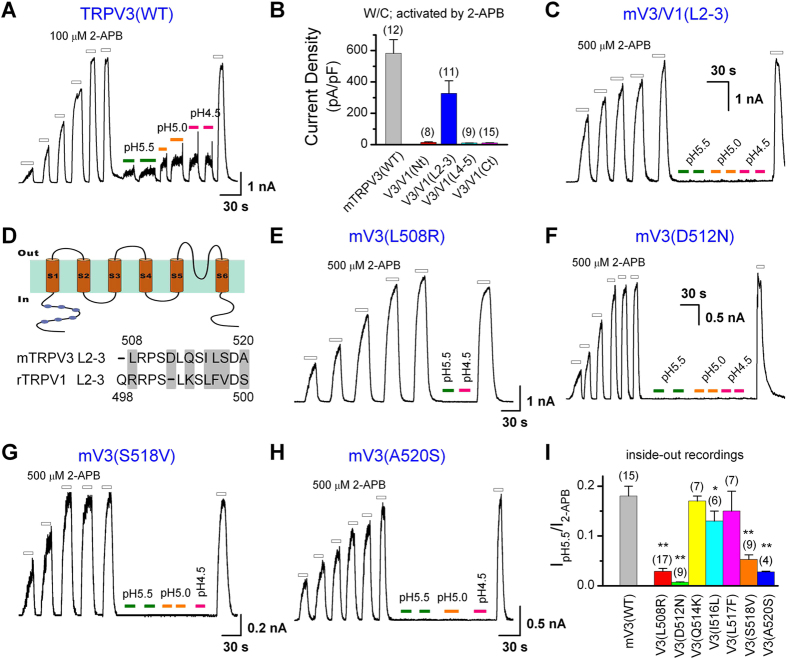
Residues in the linker of transmembrane domains 2 and 3 (L2–3) are critical for proton activation of TRPV3. (**A**) Representative inside-out recording showing that protons activated TRPV3 channel in a dose-dependent manner after sensitization by repeated applications of 100 μM 2-APB. (**B**) Summary data of current densities at −60 mV evoked by 2-APB [100 μM for wild type (WT), 500 μM for chimeras] obtained by whole-cell recordings. Only cells that expressed V3/V1(L2–3) showed response. Mouse TRPV3 mutants with the N terminus (Nt), linker of transmembrane domains 4 and 5 (L4–5), and the C terminus (Ct) swapped by the cognate segments of rat TRPV1, respectively, were not functional. (**C**) Intracellular protons failed to induce current in patches excised from cells that expressed mV3/V1(L2–3), a mouse TRPV3 mutant with the linker for transmembrane domains 2 and 3 replaced by the corresponding segment of rat TRPV1. The chimeric channel still responded to stimulation by 2-APB. (**D**) *Top*, putative membrane topology of a single TRPV3 subunit. *Bottom*, an amino acid alignment of the linker 2-3 between TRPV3 and TRPV1, with the different residues shaded in grey. (**E–H**) Representative inside-out recordings showing that protons failed to activate mV3(L508R) (**E**), mV3(D512N) (**F**), mV3(S518V) (**G**), and mV3(A520S) (**H**), even though the response to 2-APB was retained. (**I**) Summary of intracellular proton (pH 5.5)-activated currents, normalized to the maximum currents evoked by 2-APB after sensitization. Residues in the linker 2–3 of TRPV3 were mutated individually to the corresponding residues of TRPV1, except that D512 was substituted by N. Inside-out patches were excised from transiently transfected HEK293 cells and recorded while being held at +60 mV. *P < 0.05; **P < 0.001, different from WT. Error bars indicate SEM.

To further define the molecular determinant(s) of TRPV3 activation by cytoplasmic protons, we took advantage of the finding that only TRPV3 was activated by acid from the intracellular side and constructed a series of TRPV3/V1 chimeras with the intracellular segments of TRPV3 replaced by equivalent regions of TRPV1. The resultant chimeras were designated as V3/V1(Nt), V3/V1(L2–3), V3/V1(L4–5) and V3/V1(Ct). Upon transient expression in HEK293 cells, most chimeras did not respond to 2-APB with a current increase in whole-cell recordings, except for V3/V1(L2–3), which yielded sizable 2-APB-evoked currents, although a higher concentration of 2-APB (500 μM) was needed than for WT TRPV3 (100 μM) (Fig. 3B). We did not go after the reasons for the lack of function of other chimeras because the V3/V1(L2–3) mutant already showed the lack of proton-induced currents in inside-out patches even when the cytoplasmic pH was lowered to 4.5 (Fig. 3C). Under the same conditions, WT TRPV3 was activated, albeit very weakly, by intracellular protons in a dose-dependent manner (Fig. 3A). We thus focused on the S2–S3 linker region. Based on the sequence alignment, we found seven different amino acids between TRPV3 and TRPV1 in this region (Fig. 3D). We mutated them individually in TRPV3 to the corresponding amino acids found in TRPV1 and then performed inside-out recordings for the acid and 2-APB sensitivities of each mutant. Relative to that evoked by 2-APB, the acid-induced currents were significantly decreased in four point mutations, L508R, D512N, S518V and A520S (Fig. 3E–I), suggesting that these four residues in the S2–S3 linker of TRPV3 play a pivotal role in TRPV3 activation by protons from the cytoplasmic side.

### Proton potentiation involves a separate mechanism from proton activation of TRPV3

**Figure 4:**
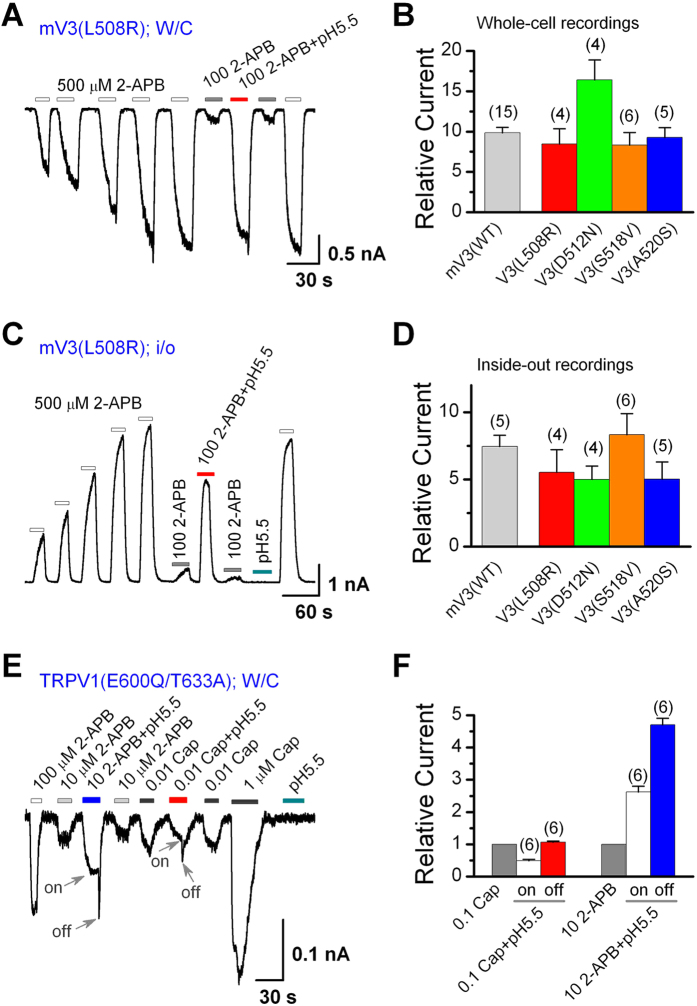
Proton potentiation of 2-APB-evoked currents remains in TRPV3 and TRPV1 mutants that show no response to acid alone. (**A**) Whole-cell currents at −60 mV for TRPV3(L508R) expressed in HEK293 cells, showing that pH 5.5 strongly enhanced the response to 100 μM 2-APB after sensitization by repeated applications of 500 μM 2-APB. (**B**) Summary of relative currents elicited by 100 μM 2-APB at pH 5.5, normalized to that by the same concentration of 2-APB at pH 7.4. WT TRPV3 and linker 2–3 mutants as described in Fig. 3 were transfected into HEK293 cells individually and whole-cell recordings performed with the cells held at −60 mV. (**C,D**) Similar to (**A**) and (**B**), but currents were recorded from inside-out patches held at +60 mV. (**E**) Whole-cell currents at −60 mV for TRPV1(E600Q/T633A) expressed in HEK293 cells, showing the responses to 2-APB, capsaicin (Cap), and low pH at indicated concentrations/levels. Results revealed that pH 5.5 enhanced the currents elicited by 10 μM 2-APB but not 0.01 μM Cap. In addition, pH 5.5 alone produced no detectable current. Note that the combination of 10 μM 2-APB or 0.01 μM Cap and pH 5.5 had a steady-state response which was followed by an off response during washout. These are marked as ‘on’ and ‘off’ by arrows. (**F**) Summary of relative whole-cell currents of TRPV1(E600Q/T633A) evoked by 0.01 μM Cap or 10 μM 2-APB at pH 7.4 and 5.5, including both the ‘on’ and ‘off’ currents at pH 5.5. Numbers of cells are shown in parentheses. Error bars represent SEM.

To evaluate whether the four residues are also critical for the proton potentiation of 2-APB-evoked responses of TRPV3, we performed both whole-cell and inside-out recordings of the mutant channels with weak 2-APB stimulation (100 μM) at pH 7.4 and 5.5, following sensitization by repeated applications of 500 μM 2-APB. In both configurations, the low pH greatly increased the responses to 2-APB, similar to that observed with the WT TRPV3 channel. Figure 4A,C show examples for V3(L508R) while Fig. 4B,D show summary data of proton potentiation of 2-APB response for all mutants tested. These results suggest that ablation of proton activation does not disrupt the potentiation effect of protons on 2-APB activation of TRPV3 channels, implying separate mechanisms between activation and potentiation of TRPV3 by intracellular protons.

We have shown earlier that in inside-out patches, although intracellular acidification did not activate TRPV1, it potentiated the response to 2-APB, but not that to capsaicin. This, together with the findings that protons potentiated 2-APB-evoked currents via a different mechanism from acid-induced activation of TRPV3 and protons also potentiated 2-APB-evoked TRPV2 currents, suggests the presence of a ligand (2-APB)- rather than channel type-dependent mechanism for the proton potentiation. However, since WT TRPV1 is activated by extracellular protons^33^, we were unable to test whether the ligand specificity also holds in the whole-cell configuration for this channel when acid was applied from the extracellular side. To overcome this problem, we constructed a double TRPV1 mutant, E600Q/T633A. Either E600Q or T633A alone had been shown to eliminate the potentiation and/or activation of TRPV1 by extracellular protons^11^,^12^. In whole-cell recordings, the double mutant indeed failed to respond to the drop of extracellular pH to 5.5 and it lost the proton-mediated potentiation of capsaicin-evoked currents. In fact, lowering the extracellular pH to 5.5 decreased the capsaicin-elicited current by nearly one-half, resulting in a rapid off response upon washout of capsaicin and the acidic solution (Fig. 4E,F), suggesting a proton-induced inhibition that could be quickly removed upon washing. Such a rapid off response was previously reported in TRPV3^18^. Importantly, the response to 2-APB (10 μM) was strongly enhanced by lowering the extracellular pH to 5.5 and the off response upon washout of 2-APB and the acidic solution was large and rapid (Fig. 4E), showing that protons greatly potentiated the effect of 2-APB on TRPV1(E600Q/T633A), even though it was confounded by the inhibitory action of the protons. Figure 4F illustrates the relative changes in peak currents elicited by capsaicin or 2-APB during (on) and immediately after the removal (off) of the pH 5.5 solution for the TRPV1 mutant. These data are consistent with those from the inside-out experiments on the WT TRPV1 and clearly demonstrate that protons selectively affect 2-APB-evoked currents in all three 2-APB activated TRPV channels. Taken together, it would appear that protons act on the ligand, 2-APB, rather than the channels to induce potentiation.

### Proton potentiation of TRPV3 is also stimulus-dependent

**Figure 5:**
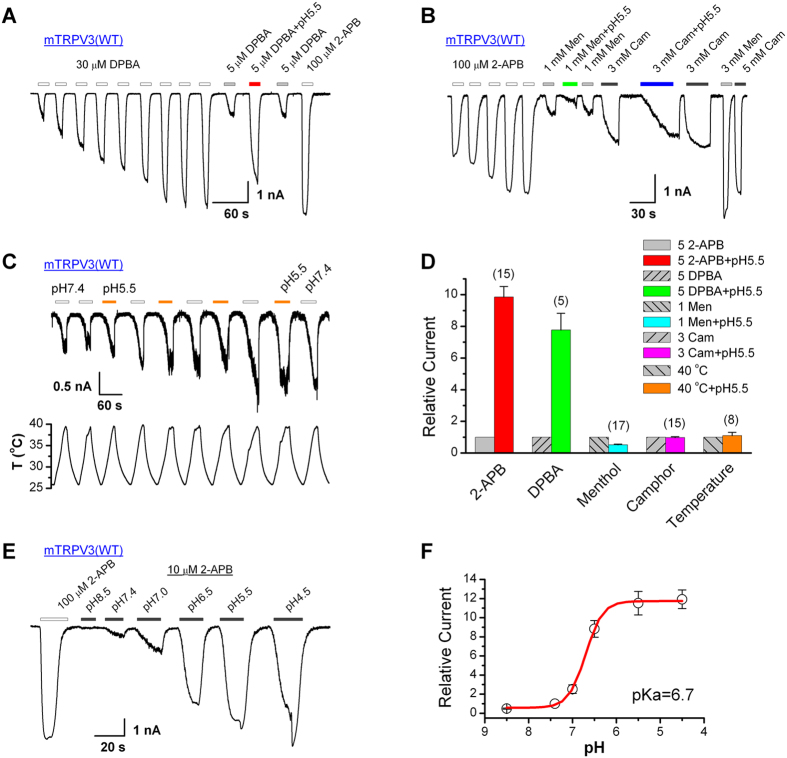
Protons potentiate TRPV3 currents in a stimulus-dependent manner. (**A**) Whole-cell recordings of TRPV3 currents evoked by DPBA. The responses to 5 μM DPBA were remarkably increased by pH 5.5 after sensitization stimulated by repeated applications of 30 μM DPBA. Holding potential was −60 mV. (**B**) Effects of protons on menthol (Men) and camphor (Cam) activated TRPV3 currents. After sensitization induced by 100 μM 2-APB, the effects of pH 5.5 on currents evoked by Men (1 mM) and Cam (3 mM) were tested. The acidic pH suppressed Men-evoked currents while slowed down the development of Cam-evoked currents without significantly altering the maximal response. Holding potential V_h_ = −60 mV. (**C**) Modulation of heat-evoked currents by extracellular protons in HEK293 cells expressing TRPV3 channels. To record heat-activated currents, bath temperatures cycled between the room temperature and ~40 °C multiple times as shown by the temperature trace below. During the heat ramps, bath pH was switched between 7.4 and 5.5. The heat-evoked currents were not significantly altered by pH 5.5. V_h_ = −60 mV. (**D**) Comparison of relative changes in current amplitude evoked by various stimuli. Peak currents activated by each stimulus in the presence of pH 5.5 were normalized to the response induced by the same stimulus at pH 7.4. (**E**) Dose dependence of pH effects on 2-APB response (10 μM). The pH (ranging from 8.5 to 4.5) effects were examined after full sensitization of TRPV3 channel by repeated stimulation with 100 μM 2-APB. V_h_ = −60 mV. (**F**) Analysis of dose-response on pH of 2-APB-activated TRPV3 currents. Figure shows averaged data fitted with the Hill equation, which generated a half-maximal pH of 6.70 ± 0.02 and n_H_ = −2.23 ± 0.17 at 10 μM 2-APB (*n* = 5). Error bars indicate SEM.

We then asked if proton potentiation of TRPV3 is also 2-APB specific. We tested three different agonists, diphenylboronic anhydride (DPBA), menthol and camphor. Among them, acid (pH 5.5) only potentiated the currents evoked by DPBA (~7-fold increase at 5 μM DPBA) while it inhibited that evoked by menthol (1 mM) by ~48% (n = 17) and slowed down the current development in response to camphor (3 mM, n = 15) (Fig. 5A,B,D). In addition, we tested whether acid alters the heat-induced TRPV3 currents. Repeated heat ramps (26–40 ^o^C) were applied with the cell perfused with solutions having alternating pH values of 7.4 and 5.5; however, the peak current amplitudes elicited by the heat are comparable between the two pH values (Fig. 5C,5D). Therefore, extracellular acidification did not increase the potency of heat to induce TRPV3 activation. Since DPBA is a structural analog of 2-APB, these data further support the notion that the proton potentiation of 2-APB-evoked TRPV3 currents results from modification of the ligand rather than its channel target.

To obtain a complete description of the pH effect on 2-APB-evoked TRPV3 activation, we tested the currents evoked by 10 μM 2-APB in solutions covering a broad pH range from 8.5 to 4.5, following channel sensitization by repeated stimulation with 100 μM 2-APB (Fig. 5E). This yielded a sigmoidal concentration-response curve with the high pH (8.5) inhibiting whereas the low pH (7.0 to 4.5) enhancing the 2-APB response (Fig. 5F). The enhancing effect reached saturation at pH 5.5. Fitting the data points with the Hill equation resulted in an EC_50_ of 6.7 ± 0.02 and an *n*_H_ of −2.2 ± 0.2. Noticeably, off responses were also detected at the low pH of 5.5 and 4.5.

### Acidic pH induces a structural switch of 2-APB

**Figure 6:**
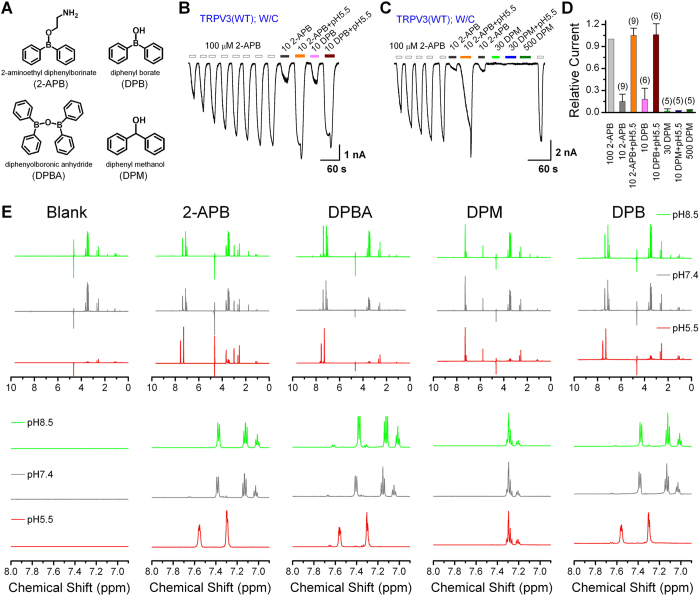
Pharmacophore of 2-APB and ^1^H-NMR (^1^H-nuclear magnetic resonance) spectra reveal structural changes of 2-APB, DPBA and DPB by acidic pH. (**A**) Structures of 2-APB, DPBA, diphenyl methanol (DPM), and diphenyl borate (DPB). (**B**) Whole-cell TRPV3 currents at −60 mV showing that the response to 10 μM DPB was potentiated by pH 5.5 after sensitization by 100 μM 2-APB. (**C**) Whole-cell TRPV3 currents at −60 mV showing the lack of response to DPM. TRPV3 channels were first sensitized by repeated applications of 100 μM 2-APB and the responses to 10 μM 2-APB were notably enhanced by pH 5.5. However, no detectable activity was evoked by 30 μM or 500 µM DPM, or the combination of 30 μM DPM and pH 5.5. At the end, 2-APB (100 μM) was applied to ensure that channels stilled functioned normally. (**D**) Summary of relative currents elicited by various combinations of compounds and pH conditions as indicated. The pH was 7.4 if not mentioned. Error bars represent SEM. (**E**) ^1^H-NMR spectra of 2-APB and its analogs under various pH environments. For 2-APB, DPBA, and DPB, the signals in the aromatic area were obviously shifted downfield at pH 5.5 as compared to pH 7.4 and 8.5. The intensities for the signals in aliphatic areas also differ between pH 5.5 and pH 7.4 or 8.5. These imply that the compounds exist in different configurations at different pH conditions. However, for DPM, there was only one configuration in solutions of various pH because of the near identical NMR signals. NMR experiments were performed at least twice with similar results. Upper panels show full spectra; lower panels show expanded spectra for the aromatic areas.

2-APB is widely used in ion channel research. Numerous studies have documented that 2-APB affects many ionic channels including inositol 1,4,5-trisphosphate receptors, store-operated calcium channels and TRP channels. As shown in Fig. 6A, several compounds, DPBA, diphenyl borate (DPB), and diphenyl methanol (DPM), are structurally related to 2-APB with the exceptions that 2-APB has an ethanolamine side chain and DPM lacks the boron that is commonly present in other analogs. Since we have shown that protons selectively enhanced the stimulatory effects of the structurally related 2-APB and DPBA on TRPV3, we next examined whether protons also potentiate the effects of the other two structural analogs, DPB and DPM. In cells sensitized by repeated stimulation of 100 μM 2-APB, the currents evoked by 10 μM DPB at pH 5.5 was significantly larger than that at pH 7.4, amounting about 5-fold increase by the acidic pH (Fig. 6B,D). However, no detectable current was elicited by DPM either at pH 7.4 (30 or 500 μM) or pH 5.5 (30 μM only) (Fig. 6C,D), suggesting that the boron was critical for TRPV3 activation by this group of compounds.

To understand how acidic pH affects the structures of these 2-APB analogs, we performed ^1^H-NMR experiments to examine their structural features at different pH (pH = 8.5, 7.4 or 5.5). As shown in Fig. 6E, the hydrogen atoms in the aromatic areas of 2-APB, DPBA, and DPB were shifted downfield while the aliphatic areas mainly had just changes in signal intensities between the structures obtained at pH 5.5 and those obtained at pH 7.4 or 8.5. In fact, the structures obtained at pH 7.4 and 8.5 were quite similar. The NMR results suggest marked structural changes between neutral and acidic pH for 2-APB, DPBA, and DPB, but not for DPM. The very similar NMR spectra of 2-APB, DPBA, and DPB under the same respective pH also indicate that these three molecules had almost the same configuration in solution (Fig. S2). This indicates that 2-APB and DPBA may be unstable in solution and easily hydrolyzed to DPB, an assessment supported by a mass spectrometry analysis, which showed that all three compounds gave rise to a negatively charged molecule of ~182 g/mole (Fig. S3B-S3D), the molecular weight of DPB. In contrast to the boron-containing analogs, DPM maintained one single configuration in all three solutions of different pH, as shown by the NMR signals (Fig. 6E). Hence, DPB exhibits a pH-dependent configuration switch, which may underlie the observed proton potentiation of TRPV1–3 channel function and explain the ligand-specificity of this peculiar regulation on 2-APB activated TRPV channels. Our data reveal the ability of 2-APB to switch between different configurations in response to environmental pH changes, which may affect its function as a TRPV activator.

Most likely, the acid-induced configuration switch involves either an addition of a proton or a loss of hydroxyl (OH^−^) group from the compound. Being a Lewis acid, the boron compound can accept a hydroxyl (OH^−^) group from water in neutral to alkaline conditions, but will lose it in the acidic solution due to competition by the increased free proton levels. This change in OH^−^ binding should transform the boron from a tetrahedral structure with four sp3 hybrid orbitals to a triangular planar structure with three sp2 hybrid orbitals and an empty p orbital. Thus, the acidification alters not only the orientation of the phenol rings but also the bond length between the boron and each phenol ring.

### Acidification of 2-APB strengthens its inhibitory effects on TRPM8

**Figure 7:**
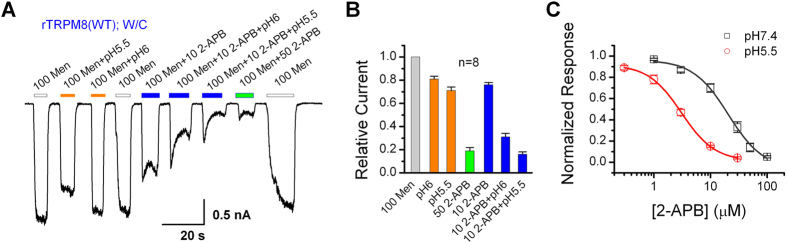
Protons enhance the inhibitory effects of 2-APB on TRPM8 channels. (**A**) Inhibition of TRPM8 by 2-APB. Representative whole-cell currents at −60 mV recorded from TRPM8-expressing HEK293 cells, showing that acid (pH 5.5 and 6.0) potentiated the inhibitory effect of 2-APB on menthol (Men, 100 μM)-evoked currents. Note, acid alone only caused weak inhibition (~19% and ~29% at pH 6.0 and 5.5, respectively), but it strongly enhanced the inhibition by 10 μM 2-APB with slow kinetics. (**B**) Summary of relative currents elicited by 100 μM Men at pH 7.4 (not indicated), 6.0, or 5.5 in the absence or presence of 2-APB as indicated. The steady-state response was normalized to the peak current evoked by 100 μM Men for each cell. (**C**) Concentration-response to 2-APB at pH 7.4 and 5.5 for currents elicited by 100 μM Men. Data were fitted by the Hill equation and yielded: IC_50_ = 20.53 ± 3.28 μM and n_H_ = 1.41 ± 0.28 for pH 7.4 (*n* = 8) and IC_50_ = 3.07 ± 0.09 μM and n_H_ = 1.49 ± 0.08 for pH 5.5 (*n* = 10). Error bars indicate SEM.

Because 2-APB is widely used in ion channel studies, we next asked whether acidification also enhances the efficacy of 2-APB regulation of other channels. To this end, we carried out whole-cell recordings of menthol-evoked currents mediated by TRPM8 expressed in HEK293 cells. As shown in Fig. 7A, at the neutral pH (7.4), 2-APB inhibited the menthol-evoked TRPM8 currents strongly at 50 μM but only weakly at 10 μM. Lowering the extracellular pH from 7.4 to 6.0 and 5.5 strongly enhanced the inhibitory effect of 10 μM 2-APB from 24 ± 2% to 69 ± 3% and 84 ± 2%, respectively (Fig. 7B). Moderate inhibition of the menthol-evoked TRPM8 currents was also observed with the low pH solution alone, amounting ~19% and ~29% for pH 6.0 and 5.5, respectively. However, with 10 μM 2-APB and pH 5.5 together, the degree of inhibition was similar to that induced by 50 μM 2-APB at pH 7.4. Figure 7C shows that the lower pH (5.5) caused a left-shift of the concentration-response curve for 2-APB. The IC_50_ reduced by about 7-fold from 20.5 ± 3.3 μM, n_H_ = 1.4 ± 0.3, at pH 7.4 to 3.1 ± 0.1 μM, n_H_ = 1.5 ± 0.1, at pH 5.5. Thus, the potency of 2-APB on inhibiting TRPM8 was also enhanced by the acidic pH, demonstrating that the proton modification of 2-APB structure may strongly impact its effects on a large number of ion channels and other protein targets.

## Discussion

Tissue acidosis is an important contributing factor to pain and inflammation^34^. Ion channels are critically involved in acid sensing under physiological and pathophysiological conditions^34^. The initial functional characterizations of both human and murine TRPV3 had indicated that the channel was not activated by low pH^17^,^20^,^21^. In accordance with these studies, we did not detect TRPV3-like activity in response to extracellular acidification in TRPV3-expressing HEK293 cells either by whole-cell patch-clamp recording or the microplate-based fluorescence Ca^2+^ assays. However, we obtained acid-induced, albeit small, TRPV3 currents under the inside-out configuration (Figs 2B,3A). This confirms the results recently published by another group that intracellular protons activate TRPV3^18^. It has also been shown that TRPV3 currents were strongly potentiated by stimulation of G_q/11_-coupled receptors via activation of phospholipase C (PLC)^24^. This could occur through PLC-mediated breakdown of PI(4,5)P_2_, which is thought to be inhibitory to TRPV3 function^35^, and/or through generation of protons, a by-product of PI(4,5)P_2_ hydrolysis^36^. However, in contrast to the previous study which showed sizeable proton-evoked currents in inside-out patches with a pH_1/2_ of ~6.1^18^, we found the acid-induced currents to be difficult to detect at pH values > pH 5.5 and very small, equivalent to 12 ± 3% (n = 8) of that evoked by 100 μM 2-APB (at pH 7.4) when stimulated by the pH 5.5 solution alone. This explains the inability of extracellular acid to elicit TRPV3 whole-cell currents, as extracellular acidification to pH 5.5 only brought down intracellular pH to ~6.15 in TRPV3-expressing cells (see Fig. 2A2).

Previously, an N-terminal residue, H426, was shown to be partially involved in intracellular proton sensing of TRPV3, but additional residues were also suggested^18^. We identified four residues located in the intracellular loop between S2 and S3 transmembrane regions of TRPV3 to be critical for channel activation by intracellular protons. The mutations in the S2–S3 loop resulted in near complete loss of intracellular proton-evoked TRPV3 currents, indicating the importance of this loop in TRPV3 gating by cytosolic protons. However, these residues do not account for proton potentiation of 2-APB response, a phenomenon common to all three TRPV channels.

The sites of 2-APB action have been determined to involve H426 at the N-terminus and R696 at the C-terminus of TRPV3^19^. Therefore, the ligand most likely acts from the cytoplasmic side to trigger channel activation. The proton potentiation occurred no matter 2-APB in the acidic solution was applied from the extracellular or intracellular side and it did not matter whether the channel being examined was TRPV1, TRPV2, or TRPV3, with potency order following the same sequence as that shown for 2-APB added at the neutral pH^29^. The potentiation of TRPV1 response to 2-APB by extracellular protons was detected after disruption of the critical residues involved in proton sensing (Fig. 4E,F), similar to the application of acidified 2-APB to the cytoplasmic side of the WT TRPV1 (Fig. 2F,G). Therefore, it is unlikely that the proton modification facilitated the penetration of 2-APB through plasma membrane. Our data that neutralization of negatively charged residues at the extracellular side of either TRPV3 or TRPV2 failed to abrogate 2-APB activation and the proton effect are also consistent with the lack of extracellular effect of the acidified 2-APB. In addition, acid pretreatment at either the intracellular or the extracellular side did not enhance TRPV3 currents activated by low concentrations of 2-APB applied to the opposite side; nor did it interfere with the potentiation by acidified 2-APB (Fig. S4), suggesting that protonation of the channel per se had no impact on acid potentiation of TRPV3 responses to 2-APB. Moreover, we show that the acidified 2-APB not only displays increased potency at TRPV channels, to which it acts as an agonist, but also at TRPM8, to which it exhibits an inhibitory action. Previous studies on 2-APB inhibition of SERCA Ca^2+^ pump and connexin hemichannels also reported the facilitation by acidic pH^37^,^38^. Altogether, these results suggest that in the acidic solution, 2-APB probably adapts a structure that is more potent at these channels and pumps than in the neutral/basic environment.

Prompted by the findings that proton protentiation of TRPV1 and TRPV3 channels was ligand rather than channel specific, we examined the effect of pH on the structures of 2-APB related compounds by NMR and found that the acidic pH induces a configuration switch of 2-APB and its boron-containing analogs. It is unlikely that this structural change involves protonation of the amino group of 2-APB because 1) its high pKa value (9.6) means that the amino group should remain protonated at both neutral and acidic pH; 2) neither DPB nor DPBA contains the amino group, but both showed similar effects as 2-APB on proton-induced potentiation of channel function and modification of solution structures; and 3) the solution structures by ^1^H-NMR and mass spectrometry measurement suggest that when dissolved in solutions both 2-APB and DPBA become hydrolyzed to DPB (Fig. S3).

The two peaks at 7.55 and 7.28 ppm in the ^1^H-NMR spectra for 2-APB, DPB, and DPBA at pH = 5.5 are identical (Fig. 6E *lower*), probably representing the hydrogen (H) atoms at the *ortho*, and *meta* + *para* positions of the phenyl rings, which have a 2:3 ratio. Under neutral and basic conditions, these H atoms have different positions and display a ratio of 2:2:1 for *ortho*, *meta, para,* where the H atoms at *meta* and *para* positions do not overlap (Fig. 6E, *lower*). Therefore, it is possible that the configuration of DPB at acidic pH represents the active species for channel activation. Since the non-boron analog, DPM, exhibited no activity on the channels and did not undergo a configuration change between neutral and acidic conditions, the boron must be critical for the structural conversion. Indeed, the arylboronic acid is a Lewis acid that can accept a OH^−^ group from water to form an anionic tetrahedral configuration with the boron in the sp3 hybridization geometry. In the acidic solution, the coordinated OH^−^ group is taken by the access H^+^ to form a water molecule, leaving behind an empty orbital on the boron atom, which converts it to the sp2 hybridized geometry. The sp3 to sp2 conversion of boron represents a dramatic structural change because the sp2 coordination gives rise to a triangular planar configuration instead of the tetrahedral shape coordinated by the sp3 geometry. As a result, not only the orientations (tetrahedral to triangular planar) of the two phenol rings but also the bond length (becomes shorter) between the phenol and boron atom are changed. This explains the large shifts in the positions of H atoms seen in the ^1^H-NMR spectra. Accordingly, the triangular planar configuration coordinated by the sp2 hybridized boron at acidic pH may represent the more active form of DPB to activate TRPV3 than the tetrahedral configuration by the sp3 boron at neutral to basic pH. Since the equivalent carbon in the inactive DPM is sp3 hybridized, it is possible that the tetrahedral configuration had no activity at these channels. Then, the activity of 2-APB and related boron-containing compounds may represent the fraction of DPB in the neutral triangular planar form in a given pH, making the response highly pH dependent. This intriguing possibility warrants the search for additional analogs that remain sp2 hybridized at neutral/basic pH and functional demonstration of their efficacy on the TRPV channels.

Aside from being modified by low pH, we interestingly found that 2-APB treatment lowers intracellular pH in a TRPV3-dependent fashion (Fig. 2A). Previously, TRPV3 has been suggested to be proton permeable^18^,^39^. Therefore, the opening of TRPV3 channels by 2-APB may be responsible for the moderate drop (~0.35 pH unit with 300 μM 2-APB) of cytosolic pH. However, the bulk of pH drop inside the cell in response to extracellular acidification is independent of TRPV3 expression and appears to be additive to the 2-APB-induced intracellular pH decrease. It has been suggested that the low extracellular pH induces a significant drop of intracellular pH probably by inhibiting the H^+^ extrusion through sodium-hydrogen exchanger 1 (NHE1), commonly expressed in keratinocytes and many other cell types and critical for the regulation of intracellular pH^40^,^41^,^42^. Thus, in TRPV3-expressing cells and under conditions when the channels are active, both pathways, i.e. NHE1 inhibition and TRPV3 mediated H^+^ influx, can contribute to the cytosolic pH drop in response to extracellular acidification.

Taken together, our study suggests two independent forms of pH regulation of TRPV3 activity. One involves residues in the S2–S3 intracellular loop of the channel for direct proton activation; the other is independent of the channel protein but likely represents structural modification of the boron-containing ligand, 2-APB, commonly used to activate TRPV1, V2, and V3 channels. The intrinsic response of TRPV3 to cytosolic acid is very weak and not shared with the related TRPV1 and V2. However, the acid potentiation of 2-APB response is very robust and not only common among the three TRPV channels, but also seen as improved potency in the inhibition of TRPM8. Our ^1^H-NMR and mass spectrometry analyses revealed that 2-APB is hydrolyzed to DPB in solution and the resulting compound adapts a distinct configuration at low pH from that in neutral and basic solutions for its actions on protein targets. Our findings reveal a novel mechanism of action for 2-APB and related compounds on TRP channels and should offer new clues to the design of potent and selective drugs against these channels and other targets known to be modulated by the 2-APB related compounds.

## Materials and Methods

### Expression constructs and transfection in HEK293 cells

Full-length cDNA for murine TRPV3 was isolated and subcloned into pIRES2-EGFP, as previously described^29^. The wild-type rat TRPV1, TRPV2 and TRPM8 cDNAs were generously provided by Dr. Feng Qin (State University of New York at Buffalo, Buffalo). All mutations were made using either QuikChange^®^ XL site-directed mutagenesis kit (Agilent Technologies) or the overlap-extension polymerase chain reaction (PCR) method as previously described^43^. The resulting mutations were verified by DNA sequencing. HEK293 cells were grown in DMEM containing 4.5 mg/ml glucose, 10% heat-inactivated fetal bovine serum, 50 units/ml penicillin, and 50 μg/ml streptomycin. For electrophysiological experiments, cells were seeded in 35 mm culture dishes and transfected with the desired DNA constructs using Lipofectamine 2000 (Invitrogen) following the protocol provided by the manufacturer. Stable TRPV3 expressing cells were selected in the G418-containing medium. For intracellular Ca^2+^ measurements, cells were transfected with the desired DNA constructs in the wells of 96-well plates without pre-seeding using Lipofectamine 2000 (Invitrogen) as described^44^.

### Intracellular Ca^2+^ measurements

Transiently transfected HEK293 cells in 96-well plates were washed once with an extracellular solution (ECS) containing 140 mM NaCl, 5 mM KCl, 1 mM MgCl_2_, 2 mM CaCl_2_, 10 mM glucose, and 10 mM Hepes, pH 7.4, and then incubated in 50 μl ECS supplemented with 5 μM Fluo-4/AM, 0.02% Pluronic F-127 (Molecular Probes) and 0.1% bovine serum albumin at 37 ^o^C for 60 min. Probenecid (2 mM, Sigma) was included in all solutions to prevent the dye leakage from the cells. At the end of the incubation, cells were washed three times with ECS and placed in 80 μl of the same wash solution. Intracellular Ca^2+^ was measured using a fluid handling integrated fluorescence plate reader, FlexStation (Molecular Devices)^44^. The acidic compound solution contained 140 mM NaCl, 5 mM KCl, 1 mM MgCl_2_, 2 mM CaCl_2_, 10 mM glucose, and 30 mM 2-(N-Morpholino)ethanesulfonic acid (MES), pH 4.0. For acid stimulation, the desired volumes of the acidic solution were delivered to the sample plate by the integrated robotic 8-channel pipettor at the preprogrammed time points. The final pH of the solution was determined using a pH meter in parallel experiments after mixing ECS and the acidic solution at the same ratio. 2-APB (Cayman Chemical Co.) was dissolved at 0.5 mM in dimethylsulfoxide (DMSO) and diluted in ECS or appropriate mixtures of ECS and the acidic solution to 3*x* the desired final concentrations and delivered at a half of the sample volume to the sample plate. The Fluo-4 fluorescence was read at excitation of 494 nm and emission of 525 nm from the bottom of the plate at 0.67 Hz.

### Intracellular pH measurements in microtiter plates

Cells seeded in wells of 96-well plates were washed with ECS as above and then incubated in 50 μl ECS supplemented with 2.5 μM BCECF/AM (TEF LABS) and 0.02% Pluronic F-127 at 37 °C for 60 min. At the end of the incubation, cells were washed three times with ECS and then placed in 80 μl of the same wash solution. Intracellular pH was measured using FlexStation with alternating excitations of 440 and 490 nm and emission of 535 nm at 0.25 Hz. The acidic compound solutions had the same composition as described above, with pH adjusted to 4.0 in order to obtain final pH values of 5.5 after compound additions.

### Electrophysiological recordings

Transfected HEK293 cells were reseeded on 12 mm round glass coverslips (Warner Instruments) one day after transfection. Whole-cell recordings were performed the following day. Recording pipettes were pulled from borosilicate glass capillaries (Sutter or World Precision Instruments) to 2–4 MΩ when filled with internal solution containing (in mM) 140 CsCl, 2.0 MgCl_2_, 5 EGTA, 10 HEPES, pH 7.4. Bathing solution contained (in mM) 140 NaCl, 5 KCl, 5 EGTA, 1 MgCl_2_, 10 glucose and 10 HEPES, pH 7.4. Isolated cells were voltage clamped in the whole-cell mode using an EPC10 amplifier (HEKA Instruments Inc). Voltage commands were made from the Patchmaster program, and the currents were recorded at 5 kHz. For a subset of recordings, currents were amplified using an Axopatch 200B amplifier (Molecular Devices, Sunnyvale, CA) and recorded through a BNC-2090/MIO acquisition system (National Instruments, Austin, TX) using custom-designed software, QStudio (http://www.qinlab.buffalo.edu). Whole-cell recordings were typically sampled at 5 kHz and filtered at 1 kHz. The compensation of pipette series resistance and capacitance were taken by using the built-in circuitry of the amplifier (>80%) to reduce voltage errors. Exchange of external solutions was performed using a gravity-driven local perfusion system (Automate Scientific). Channel activators were diluted into the recording solution at the desired final concentrations and applied to the cell through perfusion. As determined by the conductance tests, the solution around a patch under study was fully controlled by the application of a solution with a flow rate of 100 μl/min or greater. All pharmacological experiments met this criterion. The acidic solutions contained (in mM): 140 NaCl, 5 KCl, 5 EGTA, 1 MgCl_2_, 10 glucose, and 10 MES, with pH adjusted to desired values by NaOH. For inside-out recordings, both the pipette solution and bath solution contained (in mM) 140 NaCl, 5 KCl, 5 EGTA, 1 MgCl_2_, 10 HEPES, pH 7.4. Excised patches were held constantly at desired potentials while compounds were repetitively applied and washed from the bath. For recordings from HEK293 cells under acidic pH conditions, the solution also contained 50 μM amiloride as an inhibitor of the native ASIC channels. Camphor was purchased from Acros (New Jersey, USA). Diphenyl borate (DPB) was synthesized by Beijing Realchem Technology Company (Beijing, China). Other chemicals were purchased from Sigma (Sigma, St. Louis, MO). Water-insoluble reagents were dissolved in either 100% ethanol or DMSO to make stock solutions and were diluted in the bathing solution at appropriate concentrations before experiments. The final concentrations of ethanol or DMSO did not exceed 0.3%, which had no effect to the currents. All experiments except those for heat activation were performed at room temperature (22–24 °C).

### Temperature control

The temperature was controlled by constant perfusion of recording solution through an inline SC-20 heater powered by a CL100 bipolar temperature controller (Harvard Apparatus, Holliston, MA). Cells were placed in a custom-made chamber, which is narrow and has a rectangular shape. The TA-29 thermistor (Harvard Apparatus, Holliston, MA) was placed near the pipette tip to monitor the actual temperature of the recording. To determine the temperature differences between the samples and the thermocouple, we used another set of temperature controller and its thermocouple was positioned as the pipette tip normally was to read the real temperature surrounding the samples. By controlling the flow rate of the solution and keeping everything constant, we found the temperature difference between the tip and the thermistor was <0.5 °C in the heating experiments. The reported temperature corresponds to the readout of the thermistor without adjustments.

### ^1^H-NMR and mass spectrometry

The solution NMR samples were prepared at the final concentration of 1 mM for the test compound in PBS at three different pH values (5.5, 7.4 and 8.5). All spectra were acquired at 298 K, in a Varian 500 MHz spectrometer. One-dimensional (1D) ^1^H NMR spectra were collected with 2-s delays using a standard Varian 1D watergate pulse sequence. Two-dimensional (2D) ^1^H NMR TOCSY (mixing time 100 ms) and NOESY (mixing time 500 ms) spectra were collected using standard Varian TOCSY and NOESY pulse sequences. All spectra were processed using NMRPipe^45^ with 1D ^1^H spectra analyzed using ACD/NMR Processor (www.acdlabs.com) while 2D ^1^H spectra analyzed using SPARKY^46^. In TOCSY, cross-peaks were potentially generated between all resonances within a spin system, while in NOESY, the integrated intensity of a given cross-peak was interpreted in terms of the distance between the two hydrogen atoms that gave rise to the peak.

For mass spectrometry, after 2-APB (1 mg), DPBA (1 mg) and DPB (1 mg) were respectively dissolved in 10 ml methyl aldehyde, the pH of each solution was adjusted to desired values with acetic acid. The molecular mass was then measured by using positive and negative ion electrospray ionization mass spectrometry (ESI-MS, Thermo Fisher Scientific Inc., Waltham, MA).

### Data analysis

Data were analyzed offline with Clampfit (Molecular Devices), IGOR (Wavemetrics, Lake Oswego, OR), SigmaPlot (SPSS Science, Chicago, IL) and OriginPro (OriginLab Corporation, MA). Unless stated otherwise, the data are presented as the mean ± s.e.m. from a population of cells (*n*). Statistical tests of significance were carried out by Student’s *t*-test, and p < 0.05 was considered statistically significant.

## Additional Information

**How to cite this article**: Gao, L. *et al.* Selective potentiation of 2-APB-induced activation of TRPV1–3 channels by acid. *Sci. Rep.* **6**, 20791; doi: 10.1038/srep20791 (2016).

## Supplementary Material

Supplementary Information
