# Supplementary material for: Selective potentiation of 2-APB-induced activation of TRPV1–3 channels by acid
Source: Sci Rep. 2016 Feb 15;6:20791. doi: 10.1038/srep20791 (PMC4753485; doi:10.1038/srep20791)
Supplement: Supplementary Information [file srep20791-s1.doc]

**Supplementary Information**

**Selective potentiation of 2-APB-induced activation of TRPV1-3 channels by acid**

**Luna Gao^1,2,5^, Pu Yang^3,5^, Peizhong Qin^2,5^, Yungang Lu^3^, Xinxin Li^2^, Quan Tian^2^, Yang Li^2^, Chang Xie^2^, Jin-Bin Tian^3^, Chengwei Zhang^4^, Changlin Tian^4^, Michael X. Zhu^3,*^, Jing Yao^2,*^**

1 Key Laboratory of Molecular Biophysics of the Ministry of Education, College of Life Science and Technology, Huazhong University of Science and Technology, Wuhan, Hubei 430074, China;

2 College of Life Sciences, Wuhan University, Wuhan, Hubei 430072, China

3 Department of Integrative Biology and Pharmacology, The University of Texas Health Science Center at Houston, Houston, TX 77030

4 Hefei National Laboratory of Microscale Physical Sciences, School of Life Sciences, University of Science and Technology of China, Hefei 230026, China

5 These authors contributed equally to this work.

**Running title:** Regulation of TRPV1-3 channels by protons

**Keywords:** TRP channel/Inflammatory/Thermal receptor/Acidosis

*Correspondence should be addressed to:

Dr. Jing Yao

College of Life Sciences

Wuhan University

Wuhan, Hubei 430072, China

Phone: 86-27-68752148

Email: [jyao@whu.edu.cn](mailto:jyao@whu.edu.cn)

Dr. Michael X. Zhu

Department of Integrative Biology and Pharmacology

The University of Texas Health Science Center at Houston

Houston, TX 77030

Phone: 1-713-5007505

Email: [Michael.X.Zhu@uth.tmc.edu](mailto:Michael.X.Zhu@uth.tmc.edu)

4 Supplementary figures

**Supplementary Figure S1. Acid potentiation of TRPV3 and TRPV2 currents was unaffected by mutations of extracellular protonatable sites.**

(A) Representative whole-cell currents at -60 mV in a HEK293 cell that expressed the mTRPV3(E610Q). After sensitization by repeated stimulation with 100 μM 2-APB at pH7.4, the cell was exposed to 30 and 5 μM 2-APB at pH 7.4 or pH 5.5 as indicated (B) Relative currents of mTRPV3(E610Q) elicited by 30 and 5 µM 2-APB at pH 7.4 and 5.5. (C) Relative currents elicited by 5 µM 2-APB at pH 5.5 normalized to that at pH7.4 for the indicated mTRPV3 (mV3) mutants, in which protonatable residues in the putative extracellular side were changed individually to neutral ones. No significant difference was noticed between the wild type TRPV3 and the mutants. (D-F) Similar experiments performed for rTRPV2 with 300 µM 2-APB and pH 6.0 or 5.5. Note the mutations of protonatable residues in the putative extracellular side did not change acid potentiation of TRPV2. Numbers of cells are indicated in parentheses; error bars represent SEM.

**Supplementary Figure S2.** **2D ^1^H-NMR (Two-dimensional ^1^H-nuclear magnetic resonance) spectra reveal structural changes of 2-APB, DPBA and DPB, but not DPM, in acidic pH.**

The 2D ^1^H-NMR results from NOESY (A-D) and TOCSY (E-H) analyses suggest marked structural changes of 2APB, DPBA, and DPB, but not DPM, between neutral/alkaline and acidic pH. The very similar NMR spectra of 2-APB, DPBA, and DPB under the respective pH conditions also indicate that these three molecules share almost the same conformations in the respective solutions.

**Supplementary Figure S3. Chemical reactions for DPBA, 2-APB, and DPB by changing pH.**

1. When dissolved in acidic aqueous solution, 2-APB (A1) and DPBA (A2) convert to DPB by reacting with water, and DPB is the general active species. It is likely that the free orbital on the boron atom in DPB is partially coordinated by the free electron pair from the OH^-^ group at neutral/alkaline pH, and when immersed in acidic solution, the coordinated OH^-^ group is protonated, leaving behind an empty orbital on the boron atom (A3). (B-D) Negative ion ESI-MS of 2-APB (1mg), DPBA (1mg) and DPB (1mg) in 10 ml methyl aldehyde at pH 7.4 (B1, C1 and D1) and pH5.5 (B2, C2 and D2). Positive ion ESI-MS of 2-APB (1mg) in 10 ml methyl aldehyde at pH 7.4 (B3) and pH5.5 (B4). The spectrum was acquired on the thermo Finnigan LCQ mass spectrometer (Thermo Fisher Scientific Inc., Waltham, MA). The pH of each solution was adjusted to desired values with acetic acid.

**Supplementary Figure S4. Proton-dependent potentiation of TRPV3 currents requires the pretitration of 2-APB by the acidic solution.**

(A) Current traces recorded in inside-out configuration, showing that acid (pH 5.5) potentiated the response to 5 or 30 μM 2-APB applied to the cytoplasmic side after sensitization by 100 μM 2-APB, despite that the pipette solution had a pH of 5.5 (buffered with 5 mM MES) instead of 7.4. Pre-exposure of the channel to acidic solution at the extracellular side did not alter the ability of pH 5.5 to potentiate the 2-APB response from the cytoplasmic side. Membrane patches were excised from TRPV3-expressing HEK293 cells. Holding potential V_h_ = +60 mV. (B) Summary of relative changes of peak currents. (C) Representative whole-cell currents of TRPV3 recorded with the pipette pH of pH 5.5. The responses to extracellularly applied 5 and 30 μM 2-APB were still potentiated by pH 5.5 after sensitization by 100 μM 2-APB. Intracellular acidification did not change extracellular proton-mediated potentiation of 2-APB-activated currents. The grey dotted line indicates the zero current level. Recordings were from TRPV3-expressing HEK293 cells held at -60 mV. (D) Summary plot for current increase by pH 5.5. Error bars represent SEM.

**Supplementary Figure S1**


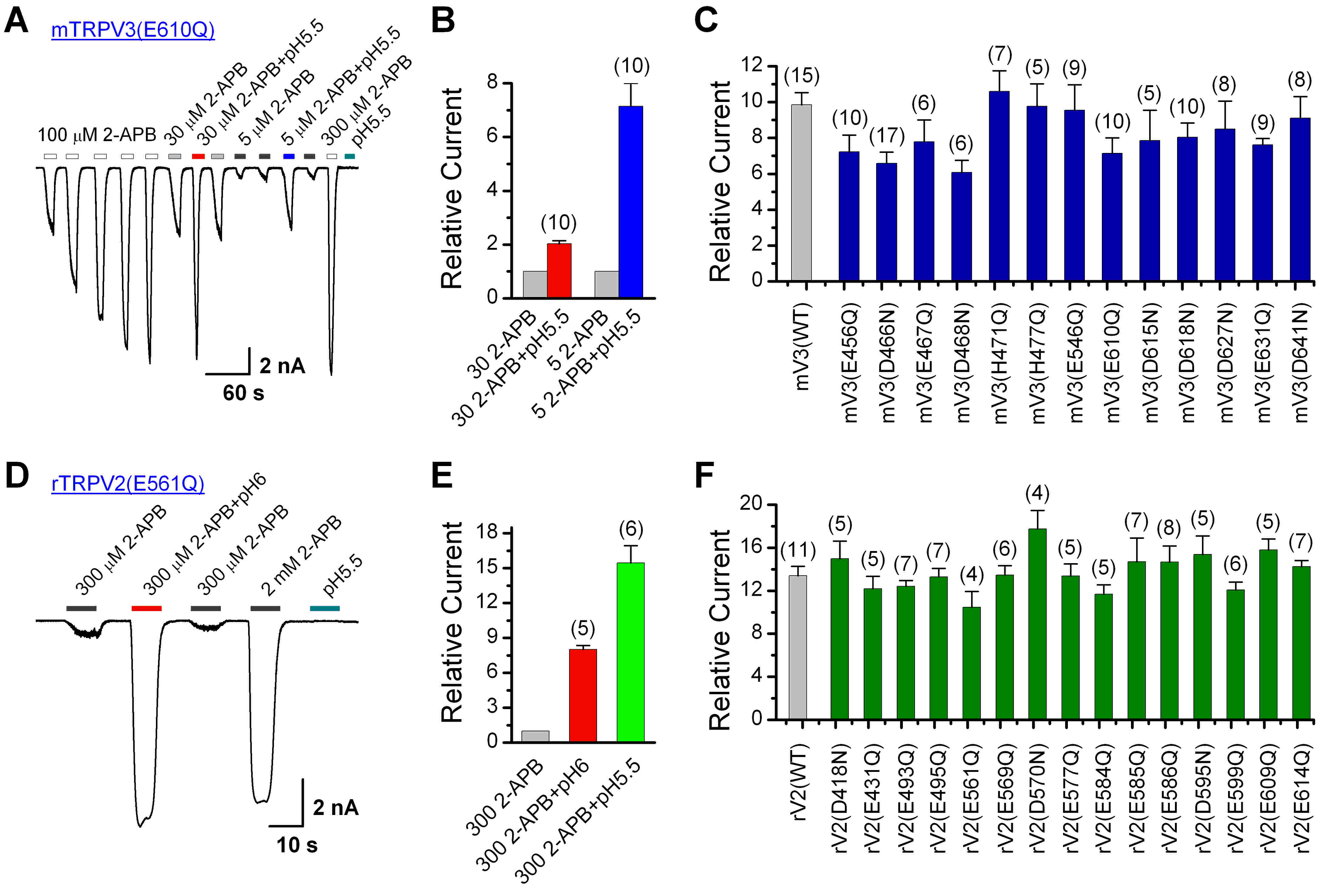


**Supplementary Figure S2**

**
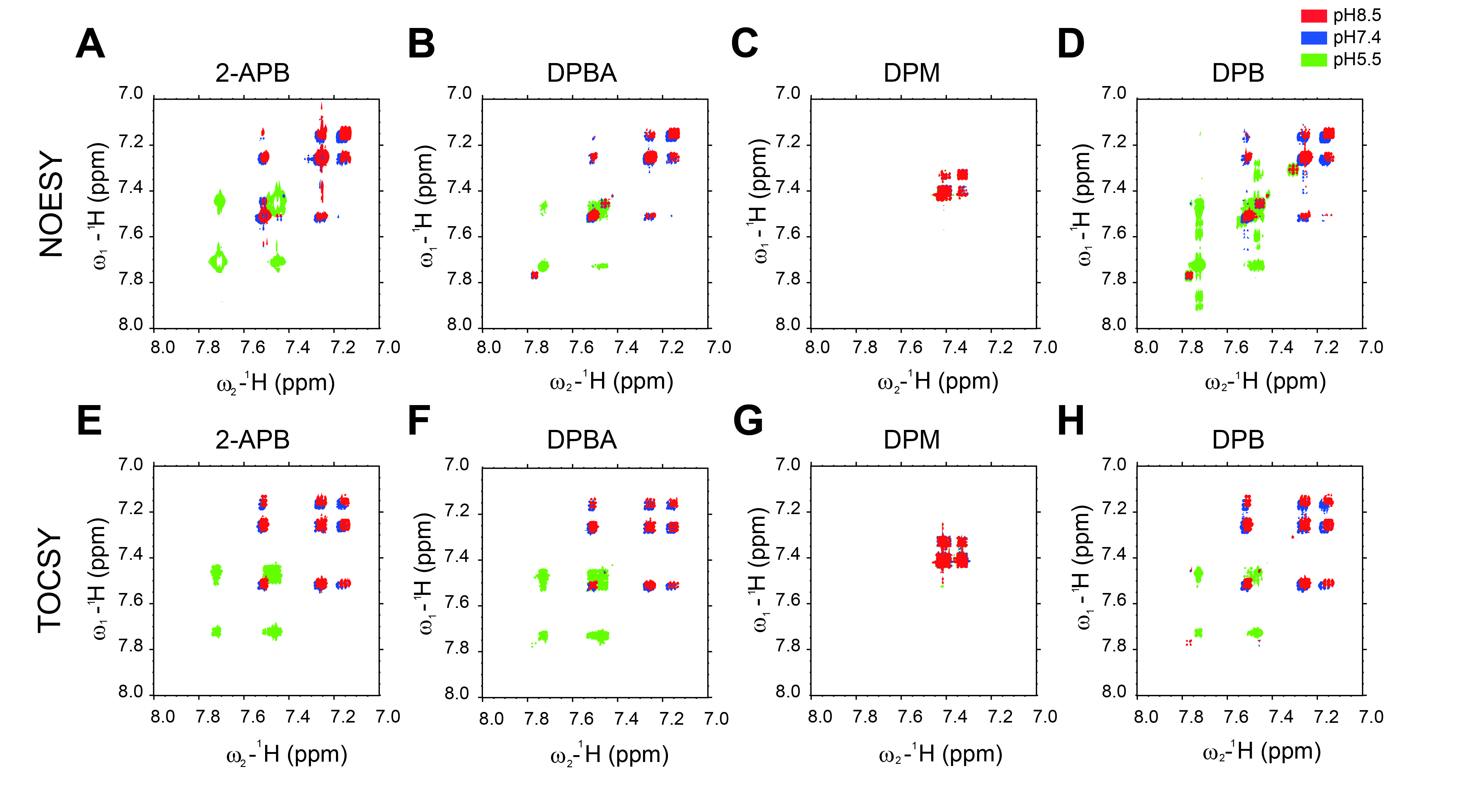
**

**Supplementary Figure S3**

**
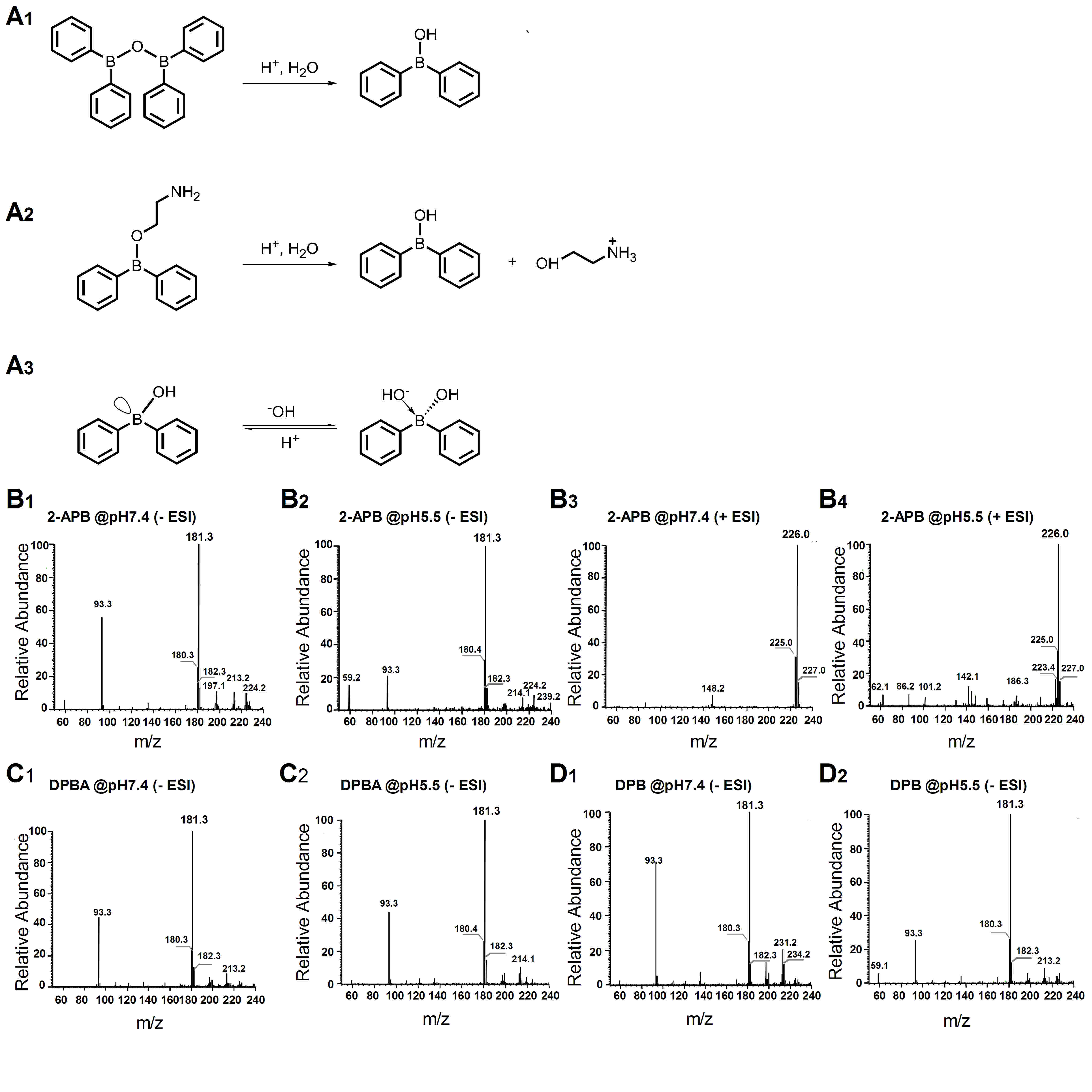
**

**Supplementary Figure S4**

**
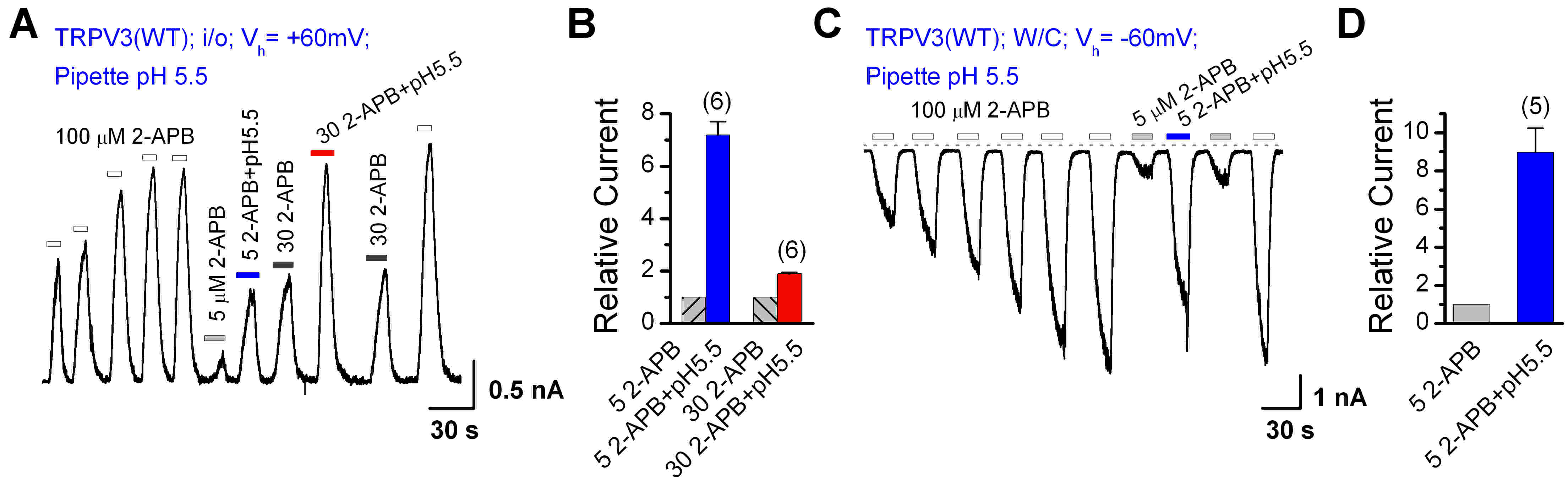
**
